# Identification and Biochemical Characterization of Four Wood-Associated Glucuronoxylan Methyltransferases in *Populus*

**DOI:** 10.1371/journal.pone.0087370

**Published:** 2014-02-11

**Authors:** Youxi Yuan, Quincy Teng, Ruiqin Zhong, Zheng-Hua Ye

**Affiliations:** 1 Department of Plant Biology, University of Georgia, Athens, Georgia, United States of America; 2 Department of Pharmaceutical and Biomedical Sciences, University of Georgia, Athens, Georgia, United States of America; Iowa State University, United States of America

## Abstract

Wood is one of the promising bioenergy feedstocks for lignocellulosic biofuel production. Understanding how wood components are synthesized will help us design strategies for better utilization of wood for biofuel production. One of the major wood components is xylan, in which about 10% of xylosyl residues are substituted with glucuronic acid (GlcA) side chains. All the GlcA side chains of xylan in wood of *Populus trichocarpa* are methylated, which is different from Arabidopsis xylan in which about 60% of GlcA side chains are methylated. Genes responsible for methylation of GlcA side chains in *Populus* xylan have not been identified. Here, we report genetic and biochemical analyses of four DUF579 domain-containing proteins, PtrGXM1, PtrGXM2, PtrGXM3 and PtrGXM4, from *Populus trichocarpa* and their roles in GlcA methylation in xylan. The *PtrGXM* genes were found to be highly expressed in wood-forming cells and their encoded proteins were shown to be localized in the Golgi. When overexpressed in the Arabidopsis *gxm1/2/3* triple mutant, PtrGXMs were able to partially complement the mutant phenotypes including defects in glucuronoxylan methyltransferase activity and GlcA methylation in xylan, indicating that PtrGXMs most likely function as glucuronoxylan methyltransferases. Direct evidence was provided by enzymatic analysis of recombinant PtrGXM proteins showing that they possessed a methyltransferase activity capable of transferring the methyl group onto GlcA-substituted xylooligomers. Kinetic analysis showed that PtrGXMs exhibited differential affinities toward the GlcA-substituted xylooligomer acceptor with PtrGXM3 and PtrGXM4 having 10 times higher *K*_m_ values than PtrGXM1 and PtrGXM2. Together, these findings indicate that PtrGXMs are methyltransferases mediating GlcA methylation in *Populus* xylan during wood formation.

## Introduction

Terrestrial plants fix about 56 billion metric tons of carbon annually, of which nearly half is stored in wood, the most abundant plant biomass [1]. Therefore, wood is an important reservoir for fixed carbon and plays a significant role in the regulation of atmospheric CO_2_ level. Furthermore, wood is a raw material vital for numerous applications, such as burning for energy, pulping and paper-making, construction, and furniture-making, and it is a renewable source for biofuel production [2]. Because of the immense role wood plays in our daily life, tremendous efforts have been devoted into understanding how wood is synthesized in order to develop molecular and genetic tools for custom-designing wood composition tailored for diverse end uses [3].

Wood is mainly composed of three wall polymers, i.e., cellulose, hemicelluloses, and lignin, the proportion of which varies among different tree species. Cellulose, consisting of linear chains of β-1,4-linked glucosyl residues, is the predominant constituent ranging from 41% to 51% in softwood and hardwood from gymnosperms and angiosperms, respectively [4]. Genes encoding cellulose synthase catalytic subunits involved in wood formation have been identified and functionally characterized in tree species [5]. Lignin is a complex polyphenolic polymer, the content of which varies from 25 to 35% in softwood and 18 to 25% in hardwood [4]. Genes encoding enzymes in the phenylpropanoid pathway leading to monolignol biosynthesis have been identified in tree species and transgenic trees with altered expression of lignin biosynthetic genes exhibit reduced lignin content and/or altered lignin composition [6].

The third major wood component is hemicellulose, which consists mainly of xylan and glucomannan. In softwood, glucomannan is the predominant hemicellulose (about 20% of wood) and xylan is only half as abundant, whereas in hardwood, xylan is the predominant hemicellulose (ranging from 20 to 35% of wood) and glucomannan is a minor component (about 3% of wood) [4]. Glucomannan is composed of β-1,4-linked glucosyl and mannosyl residues, and genes encoding mannan synthases responsible for glucomannan biosynthesis have been identified and biochemically characterized in several tree species, such as *Populus* and pine [7], [8]. Xylan from hardwood consists of a linear chain of β-1,4-linked xylosyl residues, to which 4-*O*-methylglucuronic acid (MeGlcA) side chains are attached at *O*-2 [4]. Hardwood xylan is often heavily acetylated; 40 to 60% of xylosyl residues contain acetyl groups at *O*-2 and/or *O*-3, depending on the tree species [9]. In addition, the reducing end of hardwood xylan from birch and *Populus* contains a tetrasaccharide sequence, β-d-Xyl-(1→3)-α-l-Rha-(1→2)-α-d-GalA-(1→4)-d-Xyl, that is distinct from the xylosyl backbone [10], [11]. The biosynthesis of xylan requires a suite of enzymes that are responsible for the xylan backbone elongation, side chain addition and modification, and the synthesis of the tetrasaccharide reducing end sequence [12]–[18]. Our understanding of genes involved in xylan biosynthesis during wood formation in tree species is still limited.

Early genomic studies of wood formation in poplar led to the first identification of a number of glycosyltransferase genes, belonging to families GT2, GT8, GT43 and GT47, that are potentially involved in the biosynthesis of wood components [12]. Further molecular and biochemical analyses of some of these wood-associated glycosyltransferase genes have demonstrated that family GT43 genes in *Populus* are functional orthologs of Arabidopsis GT43 genes and they form two functionally non-redundant groups responsible for the elongation of xylan backbone [13]–[15]. Several other *Populus* wood-associated glycosyltransferase genes, PoGT8D, PoGT8E/PoGT8F and PoGT47C, have been shown to be functional orthologs of Arabidopsis IRX8, PARVUS and FRA8, respectively, that are involved in the biosynthesis of the xylan tetrasaccharide reducing end sequence but their exact biochemical functions have not been revealed [11], [14], [16]–[18]. Genes responsible for glucuronic acid (GlcA) substitution, GlcA methylation, and acetylation of xylan in tree species have not been characterized. In Arabidopsis, three *GUX* genes encode glucuronyltransferases mediating GlcA substitution of xylan [19], [20] and three *GXM* genes encode methyltransferases catalyzing GlcA methylation of xylan [21], [22]. Acetylation of Arabidopsis xylan involves RWAs and ESK1 [23], [24], [25].

In this study, we performed functional analysis of four *Populus* genes encoding DUF579 domain-containing proteins that are induced by PtrWND2B, a wood-associated master transcriptional regulator controlling secondary wall biosynthesis [26], [27]. These PtrWND2B-induced genes, namely *PtrGXM1*, *PtrGXM2*, *PtrGXM3*, and *PtrGXM4*, are highly expressed in developing wood in *Populus* stems and their encoded proteins are localized in the Golgi. *PtrGXMs* are close homologs of Arabidopsis *GXMs* that have recently been demonstrated to encode methyltransferases catalyzing GlcA methylation of xylan [21], [22] and they were able to partially restore the 4-*O*-methylation of GlcA in xylan when overexpressed in the Arabidopsis *gxm1/2/3* triple mutant. Moreover, recombinant PtrGXMs exhibit a methyltransferase activity transferring the methyl group from the methyl donor *S*-adenosylmethionine to GlcA-substituted xylooligomer acceptors. These results demonstrate that PtrGXMs are wood-associated methyltransferases responsible for GlcA methylation of *Populus* xylan.

## Results

### Microsomes from *Populus* developing wood exhibit glucuronoxylan methyltransferase activity

**Figure 1:**
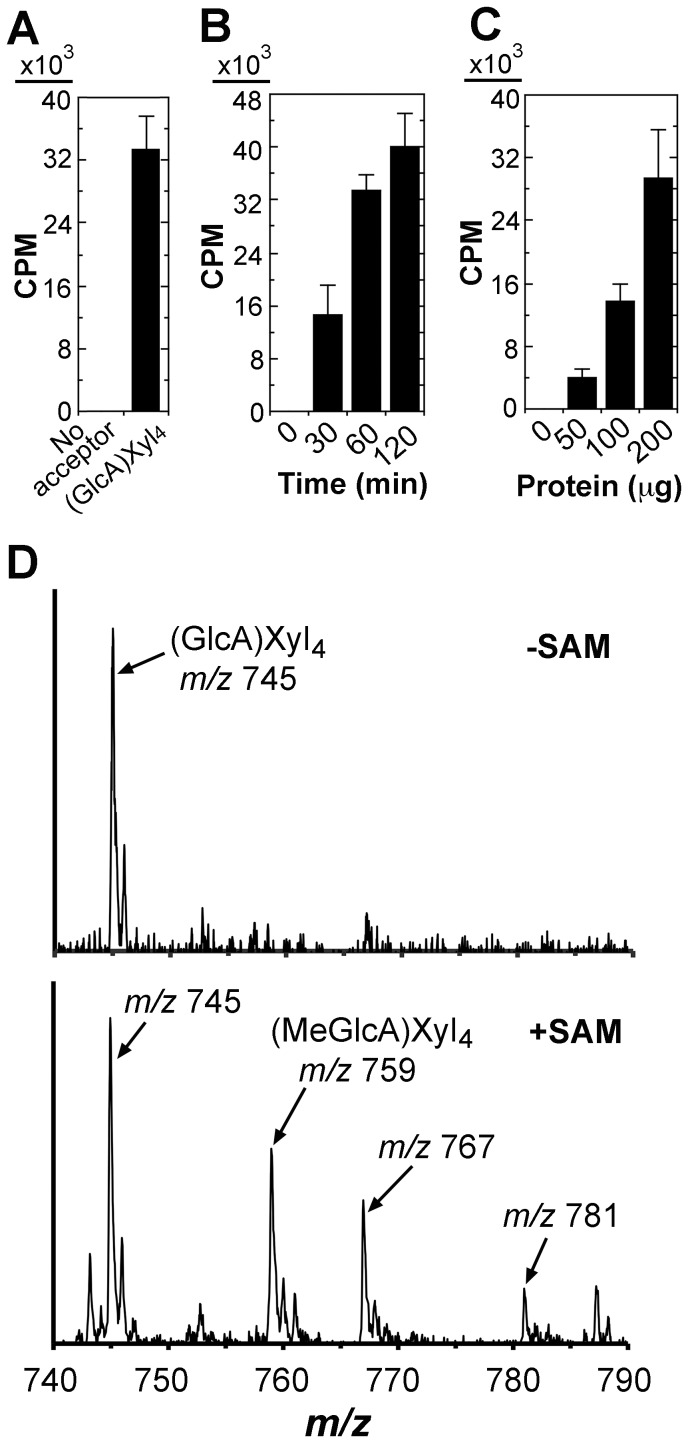
Detection of glucuronoxylan methyltransferase activity in *Populus* stem microsomes. Microsomes isolated from developing stems of *Populus trichocarpa* were incubated with the methyl donor, ^14^C-radiolabeled *S*-adenosylmethionine, and the GlcA-substituted Xyl_4_ acceptor. The methyltransferase activity (CPM) was measured by the transfer of the radiolabeled methyl group onto the acceptor. Error bars in (A), (B) and (C) denote the se of two biological replicates. (A) *Populus* stem microsomes exhibit a methyltransferase activity toward the GlcA-substituted Xyl_4_ acceptor. Microsomes (200 µg protein) were used for each reaction unless otherwise indicated. (B) Time course of the methyltransferase activity exhibited by the microsomes. (C) The methyltransferase activity increases with increasing amount of microsomes. (D) MALDI analysis of the reaction products catalyzed by the methyltransferase activity in *Populus* microsomes. Microsomes were incubated with the GlcA-substituted Xyl_4_ acceptor in the absence (top panel) or presence (lower panel) of *S*-adenosylmethionine (SAM). The ions [M+Na]^+^ at *m/z* 745 and 759 correspond to the GlcA-substituted Xyl_4_ acceptor [(GlcA)Xyl_4_] and the methylated acceptor with an increase of 14 D [(MeGlcA)Xyl_4_], respectively. The ions at *m/z* 767 and 781 are attributed to the doubly sodiated species [M+2Na]^+^ of (GlcA)Xyl_4_ and (MeGlcA)Xyl_4_, respectively. The identity of the ion at *m/z* 787 is not known.

As a first step toward molecular and biochemical characterization of methyltransferases responsible for GlcA methytlation in xylan in *Populus*, we examined glucuronoxylan methyltransferase activity in developing wood of *Populus*. Microsomes isolated from *Populus* stems were incubated with *S*-adenosyl-L-methionine, radiolabeled on the methyl group, as the methyl donor and GlcA-substituted Xyl_4_ as the acceptor. While the microsomes exhibited no methyltransferase activity in the absence of the exogenous acceptor, a high level of methyltransferase activity was detected in the presence of the GlcA-substituted Xyl_4_ acceptor (Fig. 1A). This methyltransferase activity was both time- and protein concentration-dependent (Fig. 1B, C). The reaction products were verified using matrix-assisted laser desorption ionization-time-of-flight mass spectrometry (MALDI-TOF-MS). The control reaction with the addition of the acceptor but no methyl donor exhibited an ion peak [M+Na]^+^ at mass-to-charge ratio (*m/z*) 745 that corresponds to the acceptor, GlcA-substituted Xyl_4_ (Fig. 1D). In the reaction with the addition of both the methyl donor and the acceptor, a signal peak at *m/z* 759 corresponding to MeGlcA-substituted Xyl_4_ was evident in addition to the GlcA-substituted Xyl_4_ acceptor peak (*m/z* 745) (Fig. 1D). These results demonstrate that the *Populus* stem microsomes possess a methyltransferase activity capable of transferring the methyl group from *S*-adenosyl-L-methionine onto the GlcA-substituted xylooligosaccharide acceptor.

### The expression of *PtrGXM* genes is PtrWND2-induced and wood-associated

**Figure 2:**
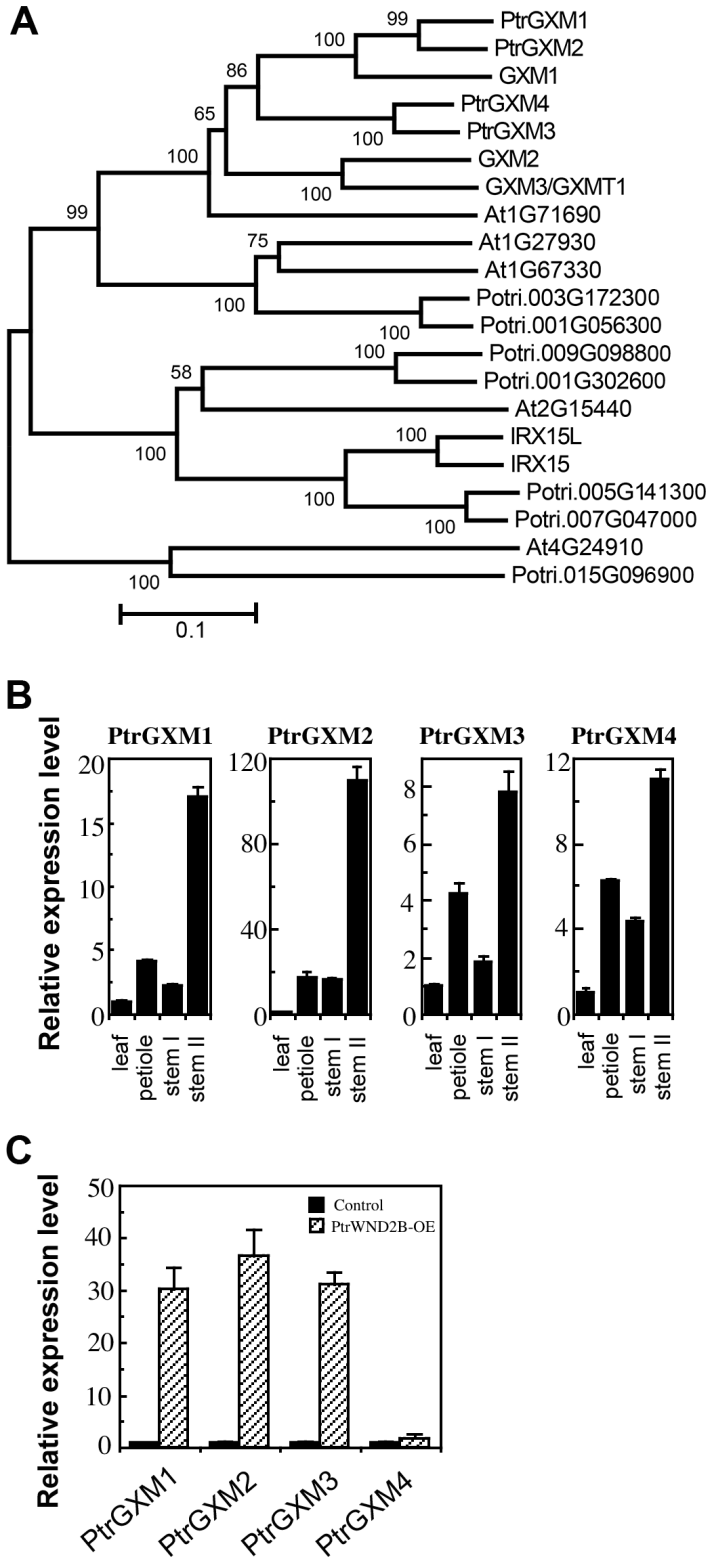
Phylogenetic and expression analyses of four *Populus GXM* genes. (A) Phylogenetic relationship of DUF579-containing proteins from *Populus* and Arabidopsis. The amino acid sequences of DUF579-containing proteins from *Populus* and Arabidopsis were aligned using ClustalW and their phylogenetic relationship was analyzed using the neighbor-joining method in MEGA5.2 [40]. Bootstrap values resulted from 1,000 replicates are shown at the nodes. (B) Quantitative PCR analysis of the expression of *PtrGXM* genes in developing leaves, petioles, stems without secondary growth (stem I), and stems with secondary growth (stem II) of *Populus*. The expression level of each gene in leaves was taken as 1. (C) Quantitative PCR analysis of the induction of expression of *PtrGXM* genes in the leaves of transgenic *Populus* plants overexpressing PtrWND2B. The control is transgenic *Populus* plants transformed with an empty vector. Error bars in (B) and (C) denote the se of three biological replicates.

**Figure 3:**
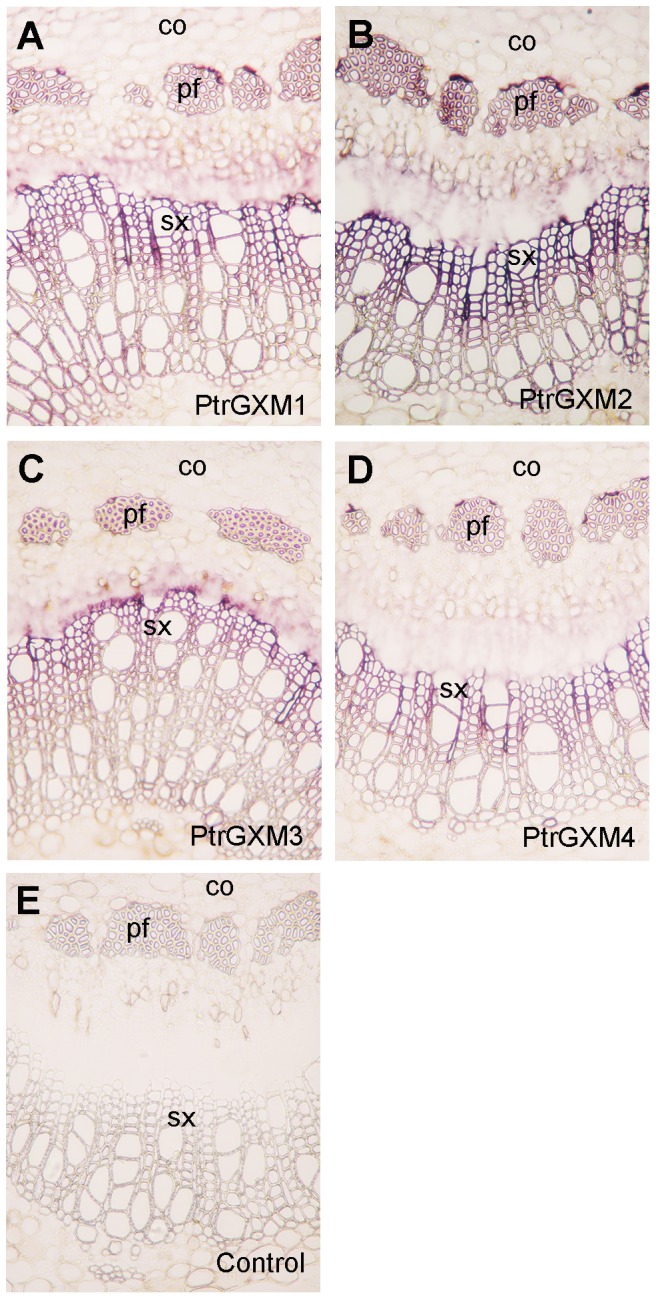
In situ hybridization analysis of *PtrGXM1*, *PtrGXM2*, *PtrGXM3* and *PtrGXM4* mRNAs in *Populus* stems. Cross sections of stems were hybridized with digoxigenin-labeled antisense probes of *PtrGXM1* (A), *PtrGXM2* (B), *PtrGXM3* (C), and *PtrGXM4* (D) or the sense probe of *PtrGXM1* as the control (E). The hybridized sections were incubated with alkaline phosphatase-conjugated antibodies and the hybridization signals are shown as purple color. co, cortex; pf, phloem fiber; sx, secondary xylem. Bar in (A) = 160 µm for (A) to (E).

To search for genes that are responsible for the above observed glucuronoxylan methyltransferase activity in *Populus* stems, we first analyzed the expression patterns of *Populus* DUF579 genes that are close homologs of Arabidopsis GXM genes. There are 11 DUF579 genes (Fig. 2A) in the *Populus* genome, of which four are closely grouped together with the Arabidopsis *GXM1*, *GXM2* and *GXM3* genes known to encode glucuronoxylan methyltransferases. These four *Populus* DUF579 genes (namely *PtrGXM1/2/3/4*) apparently fall into two pairs (*PtrGXM1/PtrGXM2* and *PtrGXM3/PtrGXM4*), each of which was likely originated from genome duplication [28]. All four *PtrGXM* genes were found to be highly expressed in *Populus* stems undergoing secondary growth (Fig. 2B). In addition, the expression of *PtrGXM1*, *PtrGXM2* and *PtrGXM3* was shown to be significantly upregulated (Fig. 2C) in transgenic *Populus* plants overexpressing PtrWND2B, which is a transcriptional master switch activating secondary wall biosynthesis during wood formation [26], [27]. *In situ* hybridization further demonstrated that the *PtrGXM* genes were predominantly expressed in cells undergoing secondary wall thickening, including developing secondary xylem and phloem fibers (Fig. 3).

### PtrGXM proteins are targeted to the Golgi

**Figure 4:**
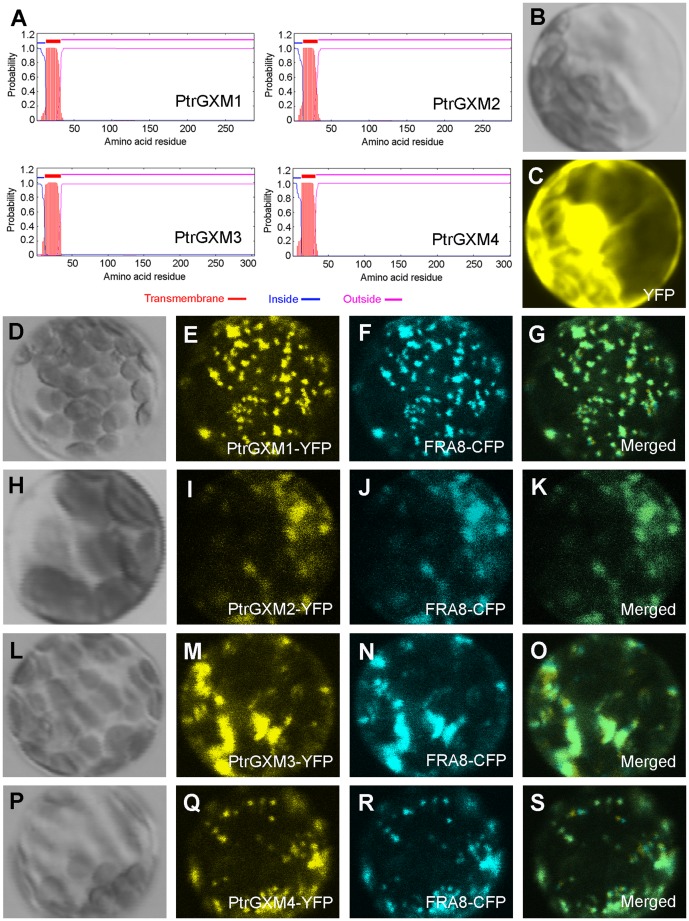
Subcellular localization of PtrGXM1, PtrGXM2, PtrGXM3, and PtrGXM4. PtrGXM proteins tagged with yellow fluorescent protein (YFP) were expressed in Arabidopsis protoplasts, and the fluorescence signals were detected with a laser confocal microscope. (A) PtrGXMs are membrane proteins with one transmembrane helix near the N-terminus as predicted by the TMHMM2.0 program. Inside, the cytoplasmic side of the membrane; outside, the noncytoplasmic side of the membrane. (B) and (C) An Arabidopsis protoplast (B; differential interference contrast image) expressing YFP alone showing the fluorescence signals throughout the cytoplasm (C). (D) to (G) An Arabidopsis protoplast (D) co-expressing PtrGXM1-YFP (E) and the Golgi-localized FRA8-CFP (F). (H) to (K) An Arabidopsis protoplast (H) co-expressing PtrGXM2-YFP (I) and FRA8-CFP (J). (L) to (O) An Arabidopsis protoplast (L) co-expressing PtrGXM3-YFP (M) and FRA8-CFP (N). (P) to (S) An Arabidopsis protoplast (P) co-expressing PtrGXM4-YFP (Q) and FRA8-CFP (R). Note the superimposition of the fluorescence signals of PtrGXM1-YFP (G), PtrGXM2-YFP (K), PtrGXM3-YFP (O), and PtrGXM4 (S) with the Golgi marker FRA8-CFP.

To further substantiate their involvement in xylan biosynthesis, we exmined whether PtrGXM proteins are targeted to the Golgi, in which xylan biosynthesis occurs. PtrGXMs are typical type II membrane proteins containing one transmembrane helix as predicted by the TMHMM2.0 program for prediction of transmembrane helices in proteins (http://www.cbs.dtu.dk/service/TMHMM-2.0) (Fig. 4A). To examine the subcellular localization of PtrGXMs, we coexpressed in Arabidopsis protoplasts yellow fluorescence protein-tagged PtrGXMs together with cyan fluorescence protein-tagged FRA8, a family GT47 glycosyltransferase known to be located in the Golgi [29]. While the control protoplasts expressing YFP alone showed fluorescent signals throughout the cytoplasm (Fig. 4B, C), the protoplasts expressing PtrGXM-YFP exhibited punctate signals, which were colocalized with the FRA8-CFP signals (Fig. 4D–S). These results demonstrate that PtrGXMs are localized in the Golgi, where xylan biosynthesis occurs.

### PtrGXMs are functional orthologs of Arabidopsis GXMs

**Figure 5:**
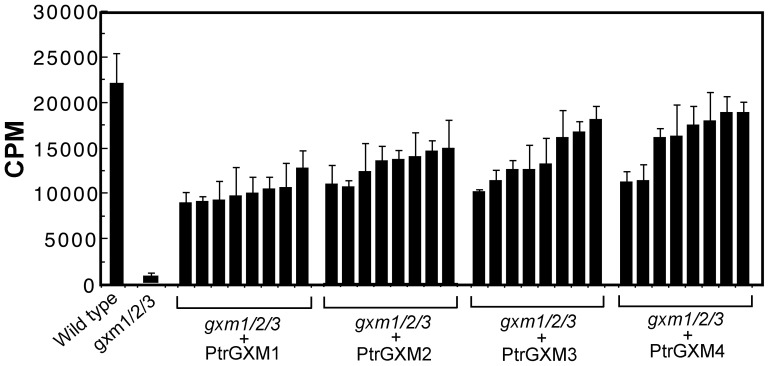
Assay of the glucuronoxylan methyltransferase activity in the Arabidopsis *gxm1/2/3* triple mutant overexpressing PtrGXMs. The full-length cDNAs of *PtrGXMs* driven by the CaMV 35S promoter were introduced into the *gxm1/2/3* mutant and the inflorescence stems of 6-week-old transgenic plants were used for microsome isolation. Microsomes were incubated with ^14^C-labeled S-adenosylmethionine and the GlcA-substituted Xyl_4_ acceptor. The methyltransferase activity (CPM) was measured by the amount of ^14^C-labeled methyl group transferred onto the acceptor. The methyltransferase activities from 8 independent transgenic lines were shown for each PtrGXM overexpression construct. Error bars denote the se of two independent assays.

The wood-associated expression of PtrGXMs and their Golgi-localization indicate that PtrGXMs most likely play a role in the biosynthesis of xylan. Considering the fact that the four PtrGXMs are phylogenetically closely grouped together with the Arabidopsis glucuronoxylan methyltransferases GXM1/2/3, we hypothesized that PtrGXMs are also glucuronoxylan methyltransferases involved in the methylation of GlcA side chains of xylan. Therefore, we tested the ability of PtrGXMs to complement the Arabidopsis *gxm1/2/3* triple mutant. The full-length *PtrGXM* cDNAs driven by the cauliflower mosaic virus (CaMV) 35S promoter were transformed into the *gxm1/2/3* mutant, and more than 64 independently transgenic lines for each construct were generated. The expression of PtrGXMs in 8 representative transgenic lines for each construct was confirmed by reverse transcription PCR analysis of PtrGXM transcripts (data not shown). Microsomes were isolated from stems of six-week-old transgenic plants and subsequently used for glucuronoxylan methyltransferase activity assays. The glucuronoxylan methyltransferase activities from 8 representative transgenic lines for each expression construct of PtrGXMs were shown in Fig. 5. It was evident that although the *gxm1/2/3* mutant exhibited a loss of glucuronoxylan methyltransferase activity, the *gxm1/2/3* lines expressing PtrGXMs showed a partial restoration of glucuronoxylan methyltransferase activity.

**Figure 6:**
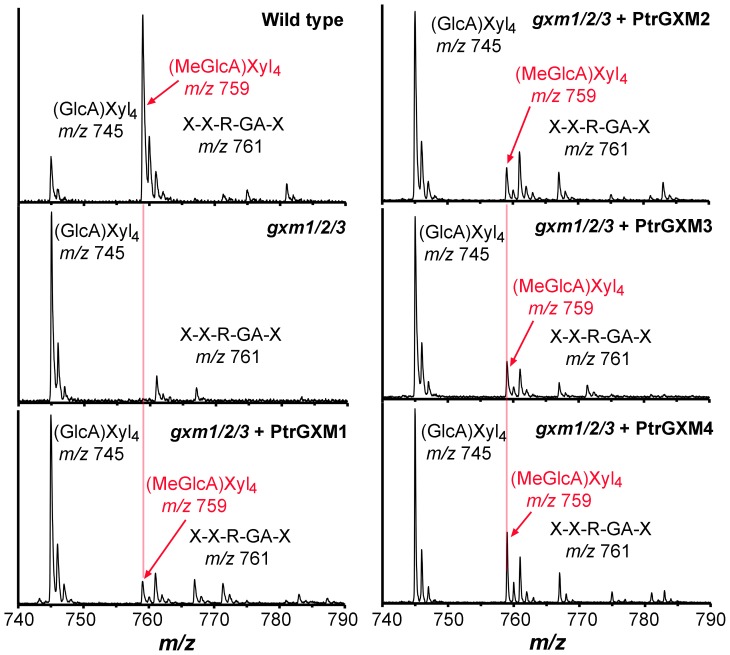
MALDI-TOF mass spectra of xylooligosaccharides generated by xylanase digestion of xylan from the *gxm1/2/3* mutant overexpressing PtrGXMs. The ions at *m/z* 745 and 759 are attributed to xylotetrasaccharides bearing a GlcA residue [(GlcA)Xyl_4_] and a methylated GlcA residue [(MeGlcA)Xyl_4_], respectively. The ion at *m/z* 761 corresponds to the xylan reducing end pentasaccharide, β-d-Xyl-(1→4)-β-d-Xyl-(1→3)-α-l-Rha-(1→2)-α-d-GalA-(1→4)-d-Xyl (X-X-R-GA-X). Note the partial restoration of the ion signal at *m/z* 759 corresponding to (MeGlcA)Xyl_4_ (red arrows) in the *gxm1/2/3* mutant overexpressing PtrGXMs.

**Figure 7:**
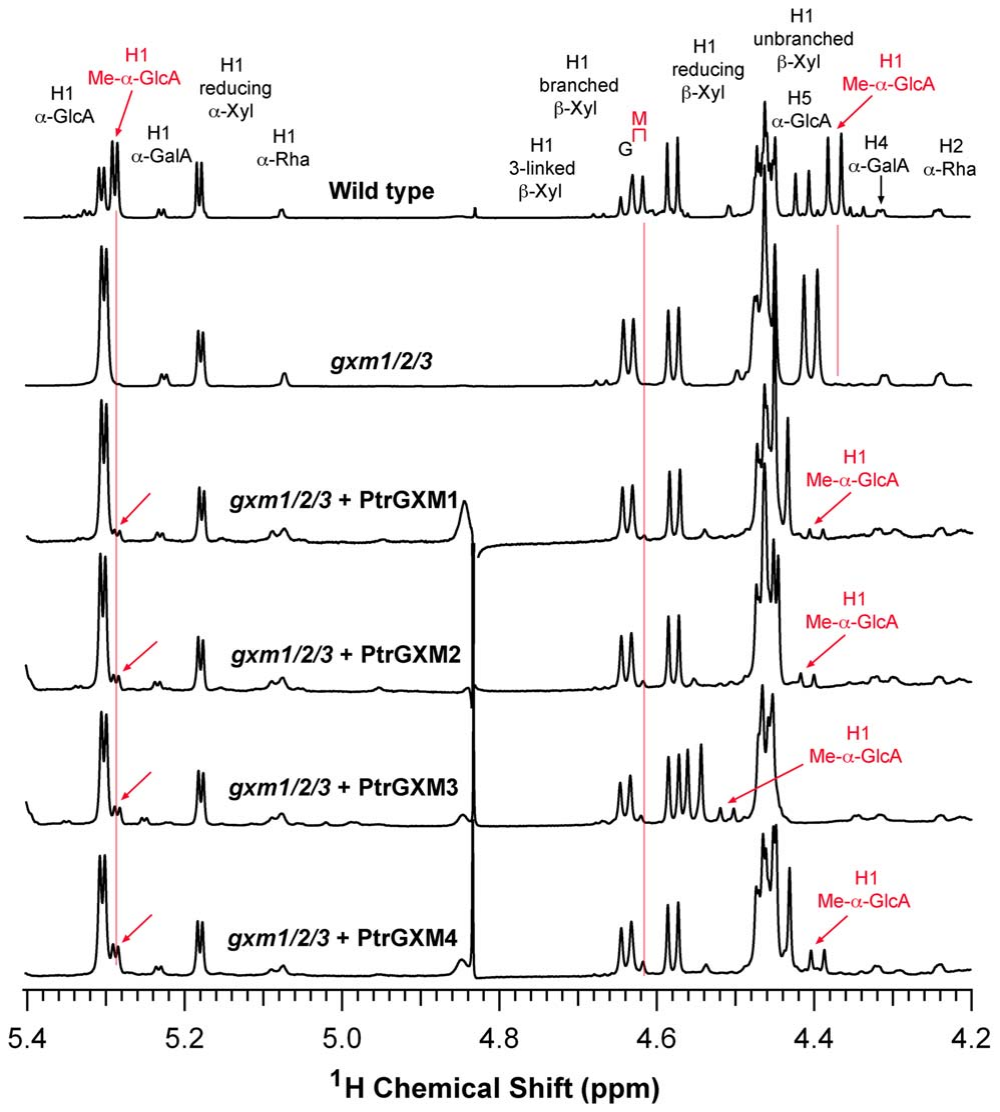
^1^H-NMR spectra of xylan from the *gxm1/2/3* mutant overexpressing PtrGXMs. Xylooligosaccharides generated by xylanase digestion of xylan were subjected to ^1^H-NMR analysis. Resonances are labeled with the position of the assigned proton and the identity of the residue containing that proton. The resonances of H1 of α-d-GalA, H1 of α-l-Rha, H1 of 3-linked β-d-Xyl, H4 of α-d-GalA, and H2 of α-l-Rha are from the xylan reducing end tetrasaccharide sequence. G and M refer to the GlcA- and Me-α-GlcA-substituted xylosyl residues, respectively. Note the partial restoration of the resonances of Me-α-GlcA (red arrows) in the *gxm1/2/3* mutant overexpressing PtrGXMs.

To find out whether expression of PtrGXMs restored the GlcA methylation of xylan in the *gxm1/2/3* mutant, we isolated xylan from the pooled stems of the 8 transgenic lines as shown in Fig. 5 for each expression contruct of PtrGXMs. The isolated xylan was digested with xylanase to generate xylooligosaccharides, which were subsequently subjected to MALDI-TOF-MS (Fig. 6). Xylooligosaccharides from wild-type xylan exhibited a prominent ion peak at *m/z* 759, which corresponds to MeGlcA-substituted Xyl_4_, and a less prominent ion peak at *m/z* 745, which corresponds to GlcA-substituted Xyl_4_. Simultaneous mutations of the Arabidopsis *GXM1*, *GXM2* and *GXM3* genes in the *gxm1/2/3* triple mutant resulted in a complete loss of the ion peak (*m/z* 759) attributed to MeGlcA-substituted Xyl_4_. As a result, the GlcA-substituted Xyl_4_ peak at *m/z* 745 was significantly elevated in the *gxm1/2/3* mutant compared with that in the wild type. Expression of PtrGXMs in *gxm1/2/3* led to a partial restoration of the ion peak at *m/z* 759 corresponding to MeGlcA-substituted Xyl_4_ (Fig. 6). The partial restoration of the GlcA methylation was further verified by ^1^H nuclear magnetic resonance (NMR) spectroscopy (Fig. 7). The wild-type xylan displayed resonances characteristic of H1 of GlcA and MeGlcA side chains at 5.31 and 5.29 ppm, respectively, and H5 of GlcA and MeGlcA side chains at 4.42 and 4.39 ppm, respectively. It also exhibited resonances at 4.63 and 4.46 ppm that are attributed to H1 of branched and unbranched backbone xylosyl residues, respectively. Xylan from the *gxm1/2/3* mutant lacked all the resonances attributed to MeGlcA side chains, including H1 of MeGlcA, H5 of MeGlcA, and H1 of MeGlcA-branched β-Xyl. Analysis of xylan from *gxm1/2/3* lines expressing PtrGXMs revealed a partial restoration of all the resonances attributed to MeGlcA side chains (Fig. 7). Integration analysis of the GlcA and MeGlcA signal peaks revealed that although the *gxm1/2/3* mutant completely lacks GlcA methylation in xylan, 8% to 21% of the GlcA side chains in xylan were methylated in *gxm1/2/3* expressing PtrGXMs (Table 1). The resonance intensities for the xylan reducing end tetrasaccharide sequence, β-d-Xyl-(1→3)-α-l-Rha-(1→2)-α-d-GalA-(1→4)-d-Xyl (H1 of 3-linked β-D-Xyl, H1 of α-L-Rha, H1 of α-D-GalA, H2 of α-L-Rha, and H4 of α-D-GalA), were unaltered in *gxm1/2/3* expressing PtrGXMs compared with the *gxm1/2/3* mutant and the wild type. Together, these results provide genetic evidence demonstrating that expression of each of the four *PtrGXM* genes in the *gxm1/2/3* mutant is able to partially restore the glucuronoxylan methyltransferase activity and the GlcA methylation in xylan, indicating that PtrGXMs are functional orthologs of the Arabidopsis GXMs catalyzing the methylation of GlcA side chains in xylan.

**Table 1:** Relative integrated values of GlcA and MeGlcA side chains in xylans from the wild type, *gxm1/2/3* and PtrGXM-complemented *gxm1/2/3* plants.

Sample	Ratio of MeGlcA relative to total GlcA and MeGlcA side chains^a^
Wild type	58.9%
*gxm1/2/3*	0%
*gxm1/2/3*+PtrGXM1	8.4%
*gxm1/2/3*+PtrGXM2	11.3%
*gxm1/2/3*+PtrGXM3	15.3%
*gxm1/2/3*+PtrGXM4	21.0%

a Ratio of MeGlcA relative to the total GlcA and MeGlcA side chains was calculated by dividing the integrated value of MeGlcA by that of the total GlcA and MeGlcA side chains based on the NMR data in Fig. 7.

### Recombinant PtrGXM proteins exhibit glucuronoxylan methyltransferase activity

**Figure 8:**
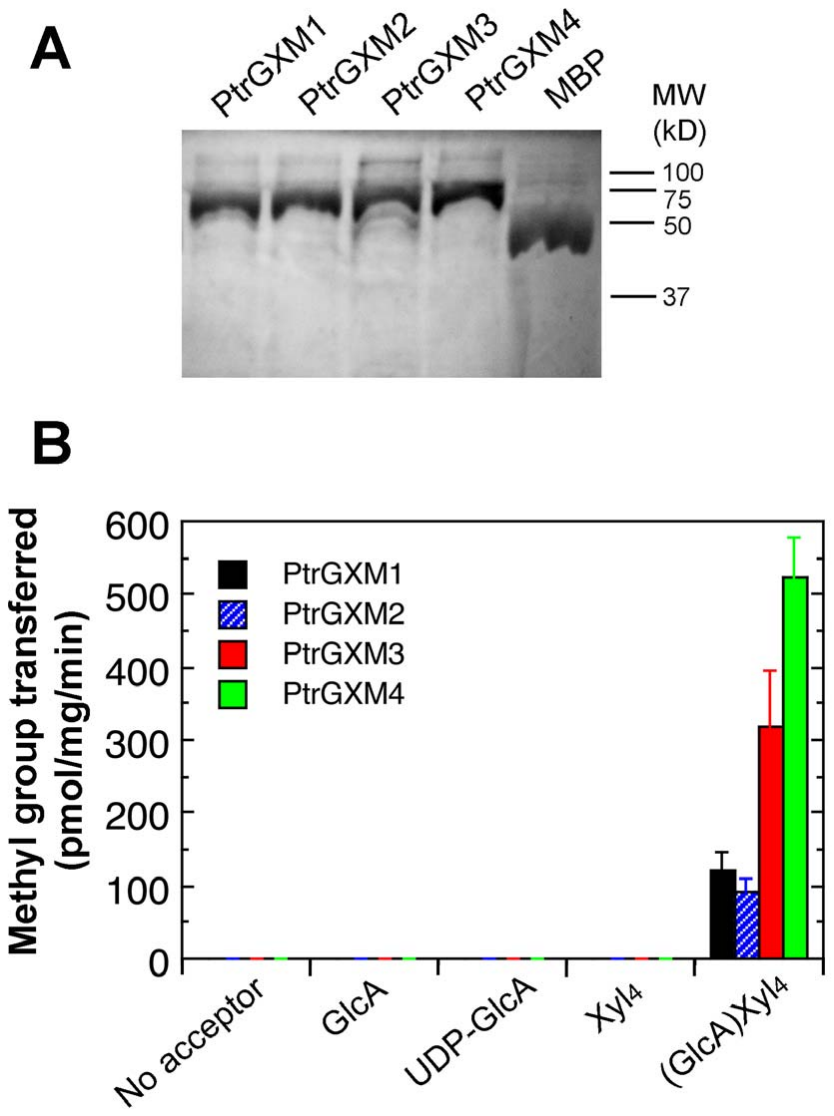
Assay of the glucuronoxylan methyltransferase activity of recombinant PtrGXM proteins. Maltose binding protein (MBP)-tagged PtrGXM proteins expressed in *E. coli* were purified with amylose resin, and used for assay of their methyltransferase activity. (A) SDS-polyacylamide gel electrophoresis detection of purified recombinant PtrGXM proteins (5 µg per lane). Shown are MBP (42.5 kD), and MBP-tagged PtrGXM1 (71.4 kD), PtrGXM2 (71.3 kD), PtrGXM3 (72.7 kD) and PtrGXM4 (72.4 kD). Molecular weight markers are indicated at right. (B) Recombinant PtrGXM proteins exhibit a methyltransferase activity that was able to transfer the radiolabeled methyl group from the *S*-adenosylmethionine donor onto the GlcA-substituted Xyl_4_ acceptor but not GlcA, UDP-GlcA or Xyl_4_. Error bars denote the se of three independent assays.

**Figure 9:**
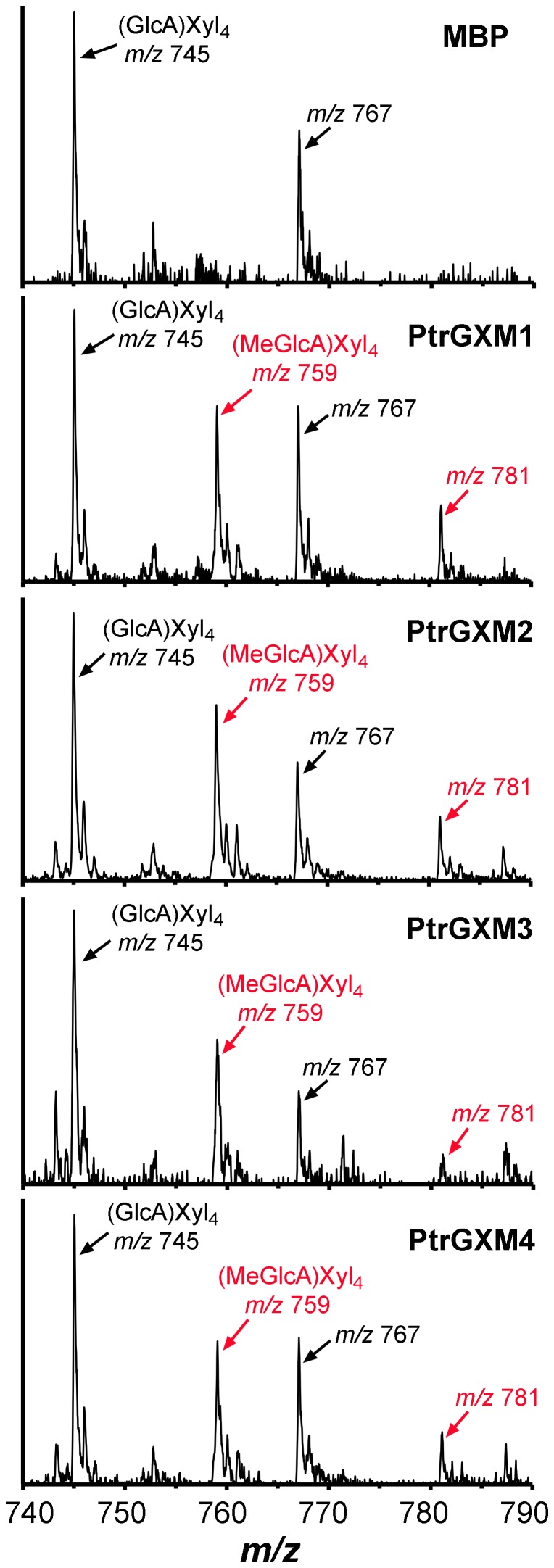
MALDI spectra of the reaction products catalyzed by PtrGXM1, PtrGXM2, PtrGXM3, and PtrGXM4 recombinant proteins. Purified recombinant PtrGXMs were incubated with the GlcA-substituted Xyl_4_ [(GlcA)Xyl_4_] acceptor and the *S*-adenosylmethionine methyl donor and the reaction products were purified and subjected to MALDI analysis. Note the appearance of a new ion peak at *m/z* 759 corresponding to (MeGlcA)Xyl_4_ (red arrows) with an increase of 14 D relative to the (GlcA)Xyl_4_ acceptor (*m/z* 745) in the products of reactions incubated with PtrGXMs but not with MBP. The ions at *m/z* 767 and 781 are attributed to the doubly sodiated species [M+2Na]^+^ of (GlcA)Xyl_4_ and (MeGlcA)Xyl_4_, respectively.

To provide definitive proof that PtrGXMs are glucuronoxylan methyltransferases, we examined the glucuronoxylan methyltransferase activity of purified recombinant PtrGXM proteins. PtrGXM1, PtrGXM2, PtrGXM3, and PtrGXM4 fused with maltose binding protein were successfully expressed in and purified from *E. coli* (Fig. 8A). Recombinant PtrGXMs were incubated with radiolabeled *S*-adenosyl-L-methionine as the methyl donor and GlcA-substituted Xyl_4_ as the acceptor. It was found that all four recombinant PtrGXMs exhibited a methyltransferase activity capable of transferring the radiolabeled methyl group onto the GlcA-substituted Xyl_4_ acceptor (Fig. 8B). In contrast, they were unable to transfer the radiolabeled methyl group onto unsubstituted Xyl_4_, GlcA, or UDP-GlcA. Purified maltose binding protein alone did not show any methyltransferase activity toward any of the substrates tested. The PtrGXM-catalyzed reaction products were further analyzed by MALDI-TOF-MS. It was evident that in addition to the prominent ion peak at *m/z* 745 attributed to the GlcA-substituted Xyl_4_ acceptor, the PtrGXM-catalyzed reaction products had another noticeable ion peak at *m/z* 759 corresponding to MeGlcA-substituted Xyl_4_ (Fig. 9). The control reaction incubated with maltose binding protein contained only the ion peak for the acceptor (*m/z* 745). These results unequivocally demonstrate that PtrGXM1, PtrGXM2, PtrGXM3, and PtrGXM4 are glucuronoxylan methyltransferases catalyzing the transfer of the methyl group onto GlcA side chains in xylan.

**Figure 10:**
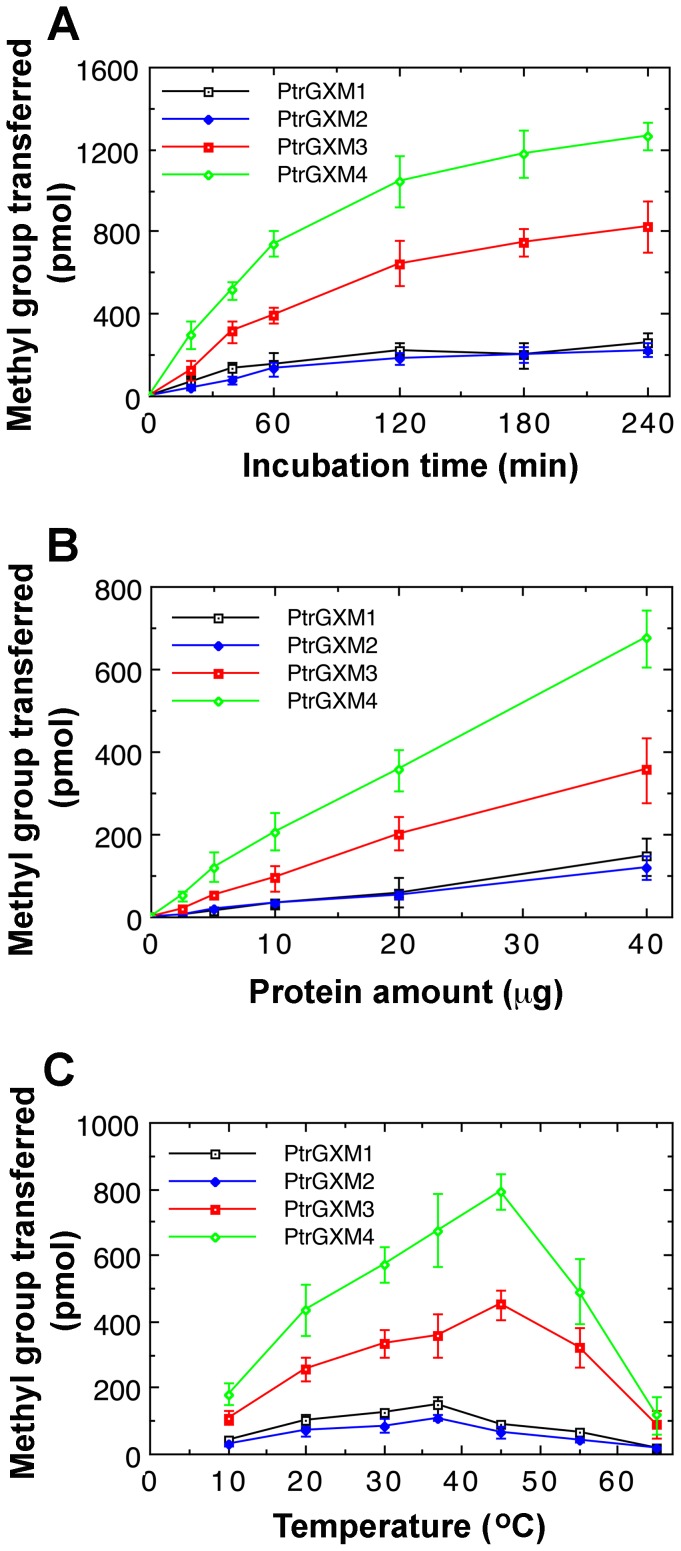
Biochemical properties of the methyltransferase activities exhibited by the recombinant PtrGXM proteins. Recombinant PtrGXM proteins were incubated with ^14^C-labeled *S*-adenosylmethionine and the (GlcA)Xyl_4_ acceptor, and the methyltransferase activity was determined by the transfer of the radiolabeled methyl group onto the acceptor. Error bars denote the se of three independent assays. (A) Time course of the transfer of the methyl group onto the (GlcA)Xyl_4_ acceptor. (B) The methyltransferase activity is protein concentration-dependent. (C) Effect of temperature on the methyltransferase activity.

**Figure 11:**
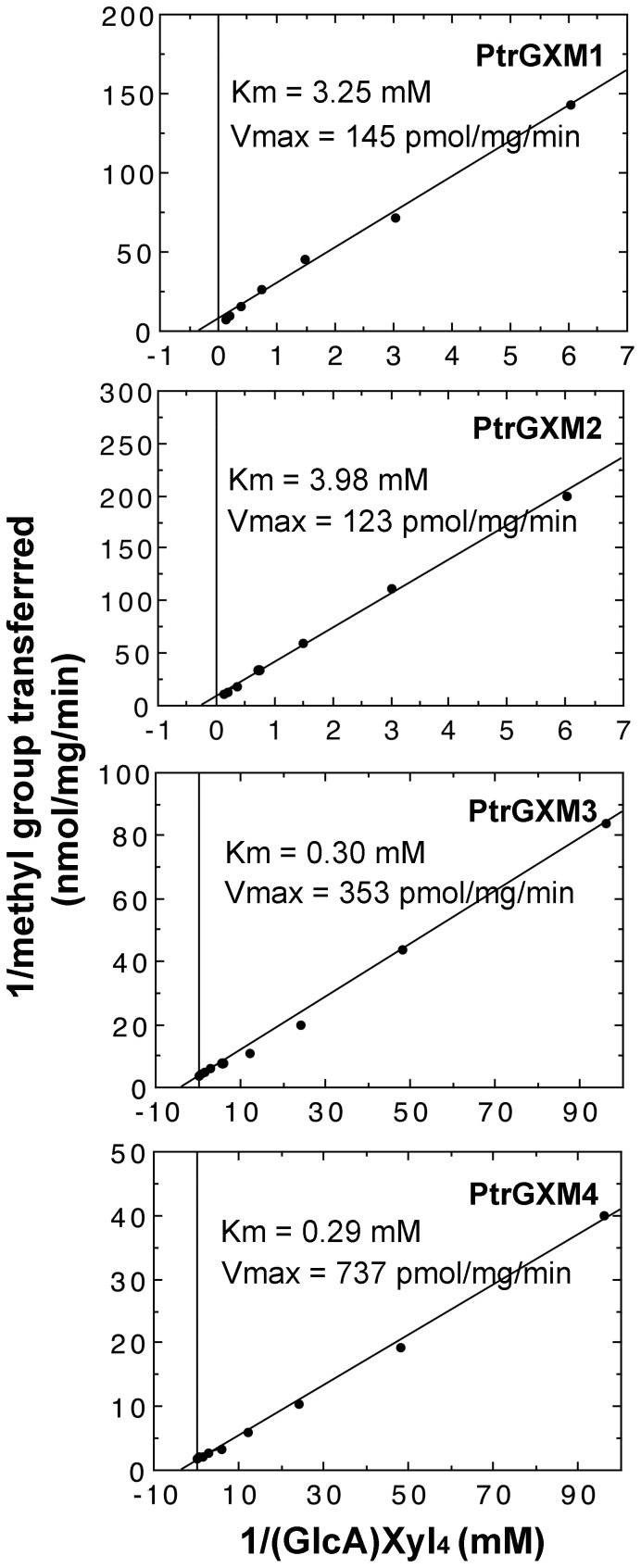
Kinetic properties of the PtrGXM methyltransferase activities. Recombinant PtrGXM proteins were assayed for the methyltransferase activity in the presence of various concentrations of the (GlcA)Xyl_4_ acceptor. The results were analyzed by Lineweaver-Burk plots to determine the *K*_m_ and *V*_max_ values.

We further analyzed the biochemical properties of the recombinant PtrGXMs. The glucuronoxylan methyltransferase activities of PtrGXMs were both time- and protein concentration-dependent (Fig. 10A, B). The optimal temperature for the reactions was 37°C for PtrGXM1 and PtrGXM2 and 45°C for PtrGXM3 and PtrGXM4 (Fig. 10C). To examine the substrate binding affinities of PtrGXMs, we investigated their kinetic properties using different concentrations of the GlcA-substituted Xyl_4_ acceptor. The *K*_m_ and *V*_max_ values were calculated by Lineweaver-Burk analysis. It was found that the transfer of the methyl group onto GlcA-substituted Xyl_4_ by PtrGXMs was dependent on the acceptor concentration (Fig. 11) with apparent *K*_m_ values of 3.25, 3.98, 0.30 and 0.29 mM and *V*_max_ of 145, 123, 353 and 737 pmol/min/mg protein for PtrGXM1, PtrGXM2, PtrGXM3, and PtrGXM4, respectively. These results indicate that PtrGXM3 and PtrGXM4 exhibit much higher substrate affinities for the GlcA-substituted Xyl_4_ acceptor than PtrGXM1 and PtrGXM2.

## Discussion

*Populus* is one of the many plant species that have been considered for use as lignocellulosic biofuel feedstocks [2]. The bulk of *Populus* feedstock is wood, which is mainly composed of cellulose (43–48%), xylan (18–28%) and lignin (19–21%) [30]. Therefore, a complete understanding of how these wood components are synthesized will be instrumental for a better utilization of this abundant feedstock for biofuel production. Previous studies have demonstrated that a number of genes, belonging to GT43, GT47, and GT8 families, are involved in the biosynthesis of the backbone and the reducing end sequence of xylan in *Populus* [11], [13]–[18]. Our current finding that four *Populus* DUF579 domain-containing proteins, PtrGXM1, PtrGXM2, PtrGXM3 and PtrGXM4, are methyltransferases that mediate GlcA methylation in xylan further enriches our understanding of the biosynthesis of xylan, the second most abundant polysaccharide in dicot wood.

Several lines of genetic and biochemical evidence demonstrate that the four PtrGXMs are wood-associated glucuronoxylan methyltransferases. First, they are expressed in developing wood undergoing secondary wall thickening. Second, the expression of *PtrGXMs* except *PtrGXM4* is activated by PtrWND2B, a master transcriptional switch regulating the secondary wall biosynthetic program during wood formation. Since there are 11 other wood-associated PtrWND homologs in *Populus* [27], it is possible that *PtrGXM4* expression is regulated by other PtrWNDs. Third, PtrGXMs are able to partially restore the deficiency in GlcA methylation in xylan when expressed in Arabidopsis *gxm1/2/3* triple mutant. Fourth, their recombinant proteins exhibit a methyltransferase activity capable of transferring the methyl group from *S*-adenosyl-L-methionine onto the GlcA-substituted xylooligomer acceptor. As PtrGXMs are unable to transfer the methyl group onto GlcA or UDP-GlcA, it is clear that the methylation of GlcA occurs after it is added onto the xylan backbone. Further biochemical analysis of PtrGXMs revealed that while PtrGXM1 and PtrGXM2 share similar biochemical properties in the temperature optimum, *K*_m_ and *V*_max_, PtrGXM3 is more similar to PtrGXM4, which is consistent with the phylogenetic analysis showing that PtrGXM1 and PtrGXM2 are a pair of genes likely originated from genome duplication, and so are PtrGXM3 and PtrGXM4. It is interesting to note that PtrGXM3 and PtrGXM4 have much higher substrate affinities and reaction rates than PtrGXM1 and PtrGXM2, indicating that the GlcA methylation in *Populus* xylan might be mainly mediated by PtrGXM3 and PtrGXM4.

Although the structure of xylan, including the xylan backbone, the reducing end sequence, the degree of acetylation and the frequency of GlcA substitution, between *Populus* and Arabidopsis is similar [11], [14], the degree of methylation of GlcA side chains differs significantly. The GlcA side chains in *Populus* xylan are all methylated, whereas only about 60% of the GlcA side chains in Arabidopsis xylan are methylated. It is currently unknown what factors account for the difference of the degree of GlcA methylation in xylan between *Populus* and Arabidopsis. Enzyme kinetic analysis of PtrGXMs revealed that although PtrGXM1 and PtrGXM2 showed *K*_m_ values (3.25 and 3.98 mM, respectively) similar to that of the Arabidopsis glucuronoxylan methyltransferase GXM3/GXMT1 (*K*_m_ = 3.85 mM) that is responsible for the bulk of the GlcA methylation of xylan [22], the *K*_m_ values of PtrGXM3 and PtrGXM4 (0.30 and 0.29 mM, respectively) were about 8 times lower. This finding indicates that PtrGXM3 and PtrGXM4 have a much higher substrate affinity than the Arabidopsis glucuronoxylan methyltransferase, which may enable a complete methylation of the GlcA side chains in *Populus* xylan during wood formation. It is also possible that the amount of GXM enzymes for GlcA methylation is much higher in *Populus* wood than in Arabidopsis secondary wall-forming cells, and therefore sufficient glucuronoxylan methyltransferase activities are available for a complete methylation of GlcA side chains in xylan in *Populus* but not in Arabidopsis.

Although expression of PtrGXMs in the Arabidopsis *gxm1/2/3* triple mutant restored the level of glucuronoxylan methyltransferase activities close to that of the wild type, the PtrGXM-complemented transgenic plants only resulted in a partial restoration of GlcA methylation in xylan. This partial complementation could be due to several possible reasons. Since multiple GXMs are expressed in cells undergoing xylan biosynthesis in both *Populus* and Arabidopsis, it is possible that the expression of one PtrGXM in the *gxm1/2/3* mutant may not be sufficient to catch up with the overall rate of xylan biosynthesis so that only a fraction of GlcA side chains are methylated. It is also possible that GlcA methylation is carried out by a protein complex containing GXMs and other xylan biosynthetic enzymes. PtrGXM sequences may have diverged from Arabidopsis GXMs to such a degree that they may not be efficiently incorporated into the Arabidopsis xylan biosynthetic enzyme complex and hence there is not sufficient GXM activity available in the complex to restore GlcA methylation during xylan biosynthesis in planta. This possibility appears to be in agreement with the observation that although expression of PtrGXMs in the *gxm1/2/3* mutant only led to low levels of GlcA methylation in xylan, the glucuronoxylan methyltransferase activity as assayed in vitro, which does not require the xylan biosynthetic enzyme complex, was restored to a much higher level.

The potential use of *Populus* as a bioenergy feedstock for biofuel production largely depends on whether it is sufficiently economical to convert cell wall polysaccharides into fermentable sugars [2]. One of the major obstacles for efficient utilization of plant biomass for biofuel production is biomass recalcitrance, i.e., resistance of cell walls to enzymatic digestion into sugars. Lignin and xylan, both of which form cross-linking networks in cell walls, contribute to biomass recalcitrance by blocking the access of cellulolytic enzymes to cellulose [31]. A reduction in the degree of GlcA methylation in xylan has been shown to enhance xylan release during hydrothermal treatment of cell walls, indicating that decreasing GlcA methylation in xylan could facilitate the conversion of biomass into fermentable sugars [22]. Our identification of four glucuronoxylan methyltransferase genes in *Populus* provides molecular tools to modify the cell wall composition of wood so that it would be more suitable for conversion into biofuels.

There exist 11 genes encoding DUF579 domain-containing proteins in the *Populus* genome (Fig. 2). Our study has established that four of them, PtrGXM1, PtrGXM2, PtrGXM3, and PtrGXM4, are glucuronoxylan methyltransferases. Four other *Populus* DUF579 genes (Potri.005G141300, Potri.007G047000, Potri.001G302600, and Potri.009G098800) fall into the same phylogenentic subgroup as Arabidopsis IRX15 and IRX15L that have also been implicated in xylan biosythesis [32], [33]. Further functional characterization of *Populus* DUF579 genes will undoubtedly provide new insights into our understanding of the biochemical mechanisms of xylan biosynthesis during wood formation in tree species.

## Methods

### Microsome isolation and methyltransferase activity assay

Microsomes were isolated from *Populus trichocarpa* stems or Arabidopsis inflorescence stems according to Kuroyama and Tsumuraya [38] and used for the methyltransferase activity assay [21]. The protein concentration in microsome preparations was measured using the Bio-Rad protein assay kit. Each reaction mixture (50 µl) contained 100 mM Tris-HCl, pH 8.0, 1 mM CoCl_2_, 0.5% Triton X-100, 2 µg/µl GlcA-substituted Xyl_4_ isolated from the *gxm2/3* double mutant, *S*-[^14^C-methyl] adenosylmethionine (0.1 µCi; American Radiolabeled Chemical), and 200 µg microsomes or 20 µg recombinant PtrGXM proteins. For MALDI analysis of GXM-catalyzed reaction products, non-radiolabeled *S*-adenosylmethionine (1 mM) was used. After 1-h incubation, the reaction mixture was passed through AG1-X4 anion exchange resin [34]. The bound MeGlcA-substituted xylooligomers were eluted with 3N acetic acid and the eluate was counted for the amount of radioactivity with a PerkinElmer scintillation counter (for radiolabeled *S*-adenosylmethionine) or subjected to MALDI analysis (for non-radiolabeled *S*-adenosylmethionine).

### MALDI-TOF MS

The reaction products from the methyltransferase activity assay or xylooligosaccharides isolated from cell walls were used for MALDI-TOF-MS according to Zhong et al. [29]. The spectrometer was operated in the positive-ion mode with an accelerating voltage of 30 kV, an extractor voltage of 9 kV, and a source pressure of approximately 8×10^−7^ torr. The aqueous sample was mixed (1∶1, v/v) with the MALDI matrix (0.2 M 2,5-dihydroxbenzoic acid and 0.06 M 1-hydroxyisoquinoline in 50% acetonitrile) and dried on a stainless steel target plate. Spectra are the average of 100 laser shots.

### Gene expression analysis

Total RNA was isolated from various organs of *Populus* or leaves of transgenic PtrWND2B-overexpressing *Populus* lines [27] with a Qiagen RNA isolation kit. They were converted into first strand cDNAs, which were used as templates for real-time quantitative PCR analysis with the QuantiTect SYBR Green PCR kit (Clontech). The PCR primers for *PtrGXM1* are 5′-actgccatatttacagctggaatg-3′ and 5′-ctactctgggcaaaagggtctgtc-3′; those for *PtrGXM2* are 5′-agaatgactgccatatatactgcc-3′ and 5′-ctactctgggcaaaaaggtctgtc-3′; those for *PtrGXM3* are 5′-tgacgaggcaccagggaggatgac-3′ and 5′-ctaaggacaaaaaggtctgcccga-3′; and those for *PtrGXM4* are 5′-tgatgaggctccagggagaatgaa-3′ and 5′-tggacaaaaaggttttcccgaact-3′. The relative expression level was calculated by normalizing the PCR threshold cycle number of each gene with that of a *Populus* actin reference gene [11]. The data were the average of three biological replicates.

### In situ mRNA hybridization

*Populus* stems were fixed in 2.5% formaldehyde and 0.5% glutaraldehyde, embedded in paraffin, and sectioned for *in situ* mRNA localization [13]. The 300-bp 3′ noncoding regions of *PtrGXMs* were used for generation of digoxigenin-labeled antisense and sense RNA probes with the DIG RNA labeling mix (Roche). The 3′ noncoding regions of *PtrGXM1* and *PtrGXM2* are highly conserved with 70% identity and those of *PtrGXM3* and *PtrGXM4* share 65% identity. There is no significant sequence identity between *PtrGXM1/2* and *PtrGXM3/*4. Dot-blot analysis showed that the *PtrGXM1* probe had a low degree of cross hybridization with *PtrGXM2* RNA and the *PtrGXM3* probe had a low degree of cross hybridization with *PtrGXM4* RNA. However, no cross hybridization was detected between the *PtrGXM1/2* probes and *PtrGXM3/4* RNA. After incubation of stem sections with the antisense or sense *PtrGXM* probes, the hybridization signals were detected by incubating with alkaline phosphatase-conjugated antibodies against digoxygenin and subsequent color development with alkaline phosphatase substrates.

### Subcellular protein localization

For subcellular localization of PtrGXMs, their full-length cDNAs were fused in frame with the yellow fluorescent protein (*YFP*) cDNA and ligated between the CaMV 35S promoter and the nopaline synthase terminator in pBI221 (Clontech). The YFP-tagged PtrGXMs together with cyan fluorescent protein (CFP)-tagged Golgi marker (FRA8) [29] were co-transfected into Arabidopsis protoplasts [35], and the fluorescence signals in transfected protoplasts were visualized using a Leica TCs SP2 spectral confocal microscope (Leica Microsystems). At least 10 fluorescence-positive protoplasts were examined and the same subcellular localization pattern was observed for each construct.

### Generation of PtrGXM overexpression lines

The full-length *PtrGXM* cDNAs were PCR-amplified, sequence-verified and then ligated into the Xba1 site between the cauliflower mosaic virus (CaMV) 35S promoter and the nopaline synthase terminator in the pGPTV binary vector [36]. The primers for *PtrGXM1* are 5′-tccaatatgaggcctaaaggccaa-3′ and 5′-ctactctgggcaaaagggtctgtc-3′; those for *PtrGXM2* are 5′-atgaggcctaaaagccagcaa-3′ and 5′-ctactctgggcaaaaaggtctgtc-3′; those for *PtrGXM3* are 5′- accatgagacccaacaaaaaccaa -3′ and 5′-ctaaggacaaaaaggtctgcccga-3′; and those for *PtrGXM4* are 5′-accatgaggtccaaaaaccaatct-3′ and 5′-ctatggacaaaaaggttttcccga-3′. An Nhe1 site was engineered at the 5′ end of each primer. The resulting PtrGXM overexpression constructs were transformed into the Arabidopsis *gxm1/2/3* triple mutant. Transgenic plants were selected on hygromycin, and their inflorescence stems were used for microsome isolation and cell wall extraction. For each construct, at least 64 transgenic lines were generated and stems of representative lines were analyzed for the glucuronoxylan methyltransferase activity.

### Cell wall extraction

Inflorescence stems of mature plants were extracted for cell walls according to Zhong et al. [29]. Cell walls were incubated with 1N KOH to release xylan, and the isolated xylan was further digested with β-xylanase M6 (Megazyme) to produce xylooligosaccharides for structural analyses [29].

### ^1^H-NMR spectroscopy

NMR spectra of the xylooligosaccharides from β-xylanase digestion were acquired at 20°C on a 600 MHz spectrometer (599.7 MHz, ^1^H) using a 3 mm cryogenic triple resonance probe [37]. All NMR samples were prepared with 100% D_2_O. For all experiments, 128 transients were collected using a spectral width of 6,000 Hz and an acquisition time of 5-seconds. The residual water resonance was suppressed by a 1-second presaturation pulse at a field strength of 40 Hz. All spectra were processed with 0.2-Hz apodization followed by zero-filling to 128 k points. The ^1^H NMR assignments were done by comparing them to the NMR spectra data for xylan structure [29], [39].

### Generation of recombinant PtrGXM proteins

The cDNAs of *PtrGXM1*, *PtrGXM2*, *PtrGXM3* and *PtrGXM4* without the transmembrane domain sequences were PCR-amplified, sequence-verified, and then ligated in frame into the BamH1 site of the bacterial expression vector pMAL, which is tagged with a maltose binding protein (MBP) (New England BioLabs). The primers for *PtrGXM1* are 5′-ccgtcttcccaggaaagtcca-3′ and 5′-ctactctgggcaaaagggtctgtc-3′; those for *PtrGXM2* are 5′-tcatcttcccaggaaagttca-3′ and 5′-ctactctgggcaaaaaggtctgtc-3′; those for *PtrGXM3* are 5′-tcatctccgagatcaaatcca-3′ and 5′-ctaaggacaaaaaggtctgcccga-3′; and those for *PtrGXM4* are 5′-tctcccagaccaaattcatcc-3′ and 5′-ctatggacaaaaaggttttcccga-3′. A BamH1 site was engineered at the 5′ end of each primer. MBP-tagged recombinant PtrGXM proteins expressed in *E. coli* were purified using amylose resin according to the manufacturer's protocol and confirmed by SDS polyacrylamide gel electrophoresis.

### Statistical Analysis

The experimental data of the quantitative PCR analysis and enzymatic activity assay were subjected to statistical analysis using the Student's *t* test program (http://www.graphpad.com/quickcalcs/ttest1.cfm), and the quantitative difference between the two groups of data for comparison in each experiment was found to be statistically significant (p<0.001).

### Accession numbers

The *Populus trichocarpa* gene locus identifiers (the *Populus trichocarpa* genome v3.0 at phytozome; http://www.phytozome.net/poplar.php) and the GenBank accession numbers for PtrGXM genes are Potri.004g226800 and KF971896, respectively, for *PtrGXM1*, Potri.003g003800 and KF971897, respectively, for *PtrGXM2*, Potri.013g102200 and KF971898, respectively, for *PtrGXM3*, and Potri.019g076300 and KF971899, respectively, for *PtrGXM4*. The *Arabidopsis* Genome Initiative locus identifiers for *Arabidopsis* GXM genes are *GXM1* (At1g09610), *GXM2* (At4g09990), and *GXM3/GXMT1* (At1g33800).
